# SLEEP QUALITY IMPROVES IN DEMENTIA CAREGIVERS USING ONLINE SELF-GUIDED ACCEPTANCE AND COMMITMENT THERAPY

**DOI:** 10.1093/geroni/igae098.2130

**Published:** 2024-12-31

**Authors:** Elizabeth Fauth, Jacob Gossner, Audrey Juhasz, Ty Aller, Michael Levin

**Affiliations:** Utah State University, Logan, Utah, United States; Utah State University, Logan, Utah, United States; Utah State University, Logan, Utah, United States; Utah State University, Logan, Utah, United States; Utah State University, Logan, Utah, United States

## Abstract

Transdiagnostic interventions treat multiple conditions by addressing common underlying mechanisms. Randomized controlled trials, systematic reviews, and meta-analyses support that Acceptance and Commitment Therapy (ACT) is a transdiagnostic mindfulness-based Cognitive Behavioral Therapy that improves anxiety, depression, pain, etc. in diverse populations. ACT efficacy is supported in dementia caregivers, typically studied via psychological or care-related outcomes. ACT improves sleep in cancer caregivers, and sleep is often disturbed in dementia caregivers. The current study examines if sleep quality improves in (N=80) dementia caregivers engaging with a caregiver-tailored six-session online, self-guided ACT program. Participants completed the Sleep Quality Scale and other assessments at T1 (pre-intervention), T2 (post-intervention) and T3 (6-week followup). A sleep-related item from a depression inventory assessed sleep restlessness. Items from a behavioral symptom inventory assessed the extent to which care receivers woke the caregiver or others in the night, and assessed caregivers’ stress appraisal of this experience. We did not hypothesize that care receivers’ wakefulness would be impacted, but hypothesized improvements in the other caregiver sleep indicators. Repeated measures ANOVA identified that caregivers improved average sleep quality [F(2,158)=7.907, p<.001; partial eta-squared =.091] and reported less sleep restlessness [F(2,156)=10.039, p<.001; partial eta-squared =.114]. Post hoc comparisons identified change occurring between T1-T2 (Intervention phase), sustained over time (T3). Care receivers’ waking behavior did not change over time (p=n.s.), but caregivers experiencing this behavior across all timepoints (N=17), reported being less bothered by it [F(2,32)=5.457, p<.01; partial eta-squared =.254]. ACT interventions offer promise in improving sleep experiences for dementia caregivers.

